# CocoaDeep: A preliminary study of the performance sensitivity to datasets of Faster RCNN, YOLO and transformer networks for cocoa pod detection

**DOI:** 10.1371/journal.pone.0351657

**Published:** 2026-07-22

**Authors:** Philippe Borianne, Frédéric Théveny, Llorenç Cabrera-Bosquet, Sabine-Karen Lammoglia

**Affiliations:** 1 UMR AMAP, CIRAD, Montpellier, France; 2 AMAP, University of Montpellier, CIRAD, CNRS, INRAE, IRD, Montpellier, France; 3 LEPSE, University of Montpellier, INRAE, Montpellier, France; 4 UFR Biosciences, University Félix Houphouët-Boigny, Abidjan, Côte d’Ivoire; 5 UMR ABSYS, CIRAD, Montpellier, France; University of Tehran, IRAN, ISLAMIC REPUBLIC OF

## Abstract

Farmers must be able to estimate their crop yields at various growth stages for effective management of their farms and to enable them to interact with cooperatives or traders as early as possible. Here we developed an AI-based cocoa pod detection method using low resolution colour images of cocoa trees on farms in Côte d’Ivoire. We compared nano and extra-large architectures of six neural networks, including Faster RCNN, Baidu’s Real-Time Detection Transformer (RTDetr), Detr-ResNet Vision Transformer (ViT), YOLOv5, YOLOv8 and YOLOv11. These networks were trained with 7,850 annotated cocoa pods on 400 low resolution images, and validated in two independent datasets: a 42 low resolution images containing 990 annotated pods, and a 100 low resolution images containing 2,400 annotated pods. The performances of the nano YOLOv8 and YOLOv11 networks were 2% higher than that of the RTDetr networks and 5% higher than that of the YOLOv5, ViT and Faster RCNN networks with an F1-score of 77% on all images and up to 90% on foreground trees. The dominance of nano architectures suggests that the extra-large architectures, which contain 20–30-times more neurons, may not have been fully trained. The study of learning performance curves showed that extra-large networks were unable to outperform nano networks, which contradicts the theory. After review, the annotated dataset was found to contain inconsistencies. The inconsistency of the training and validation data and their limited quantity restricted the objectivity of comparisons between network architectures. Finally, although the average detection performance of RTDetr for cocoa pods was only 2% lower than that of the YOLOv8 network, it was definitively excluded from the candidate models because its per-image processing time was 15–20% higher than that of YOLOv8 and YOLOv11. However, with a performance sensitivity to data of less than 0.5%, YOLOv8 Nano became the best option.

## Introduction

Estimating the yield of a crop at its various growth stages is essential for decision making on disease management, harvesting, storage, transport and marketing. Regarding cocoa (*Theobroma cacao*), a novel deep learning approach was recently presented for predicting cocoa yield using a recurrent neural network that combined spatiotemporal climatic data and statistical data on cocoa production in southwestern Nigeria [1]. However, due to a lack of suitable data, this model could not be transposed to Côte d’Ivoire, which alone accounts for >44% of world cocoa production [2], despite the fact that cocoa farming represents a major socioeconomic challenge for this country [3]. Yield estimation in that country is still based on manual counts, which are time-consuming and often hampered by major counting errors. This situation warrants the design of a low-cost, truly operational solution that could be integrated with current practices, while combining real-time identification of cocoa pods at different growth stages in natural environments with a yield estimation model based on pod counts—this would be essential for cocoa research and the cocoa industry. The main objective of this study was to propose a simple reliable AI-based method for image detection and counting of cocoa pods on low-resolution RGB mobile phone images.

In recent years, several studies have described efficient computer vision systems for fruit detection and yield estimation [4]. Fruit detection involves finding instances of fruit in an image. Computer vision systems for fruit detection involve simple pixel segmentation in terms of density or colour or more advanced machine learning methods based on a combination of colour, shape and texture features computed on images sometimes acquired by multiple or multiband sensors. Advanced machine vision systems for yield estimation use deep learning algorithms for object detection: these are being used to an increasing extent in machine vision systems for yield estimation. Among deep learning algorithms, convolutional neural networks (CNNs) have highly efficient conventional detection performance [5]. A growing number of studies have used neural networks for fruit yield estimation [6], and/or fruit detection [7, 8] and/or fruit cultivar identification [9, 10], while relying especially on Faster R-CNNs [11–13] or YOLO [14].

Numerous studies have compared diverse neural networks in terms of deep mechanisms of different families [15], as well as intra-family variations [16, 17]. Already in 2020, a comparative study of different *in situ* fruit detection networks for apples, mangoes and oranges [13] placed Faster R-CNN in third position for all fruits, with an average accuracy ~2% lower than the best results obtained using an improved Faster R-CNN. The same study ranked this network in second position for mango fruit detection, with a difference of 0.2% compared to the best results obtained with YOLOv3. More recently, a study in which a CNN model was compared to YOLO [18] for tomato detection revealed significantly superior YOLO results. A comparison between a detection transformer and YOLOv8 [19] for orange and sweet orange detection showed that the performance of these two models was relatively equivalent. The nano (n) and extra-large (x) configurations of YOLOv8 and YOLO11 were evaluated for tart cherry detection [20]. The models demonstrated robust performance. Even under conditions of high object density, YOLOv11x achieved a mAP50 of 0.92. While YOLOv8n and YOLO11n produced similar detection results, YOLOv8n had faster inference time, making it more suitable for real-time applications.

With regard to cocoa trees, most of the published studies we found concerned the detection of cocoa pod diseases [21, 22] or ripeness [23]. These studies were based on the analysis of close-up images of individual cocoa pods. They did not involve the processing of images of cocoa trees bearing multiple fruits. The few studies involving plant pathology detection on cocoa images compared different convolutional neural networks and transformers, with scores ranging from 80 to 90% [24]. [25] compared the two detection networks, i.e., U-Net and a fully convolutional network (FCN), where the latter performed much better, with a score of ~94%, i.e., nearly 2% higher than U-Net. Otherwise, [26] carried out a comparative study of different versions of Faster R-CNN and YOLOv5 detection networks, with the YOLOv5x, Faster R50FPN3x and R101C43x architectures obtaining almost identical scores of 95%.

Our work was part of the European cocoa4future project focused on the agroecological transition of cocoa farming. This project brought together many stakeholders in the cocoa sector in Côte d’Ivoire and Ghana, including more than a hundred farmers. One aspect of this project focused on studying yield estimation models. The yield estimate for a cocoa plot is often obtained by extrapolating the production estimate for a few cocoa trees chosen at random from the plot; the production of each of these cocoa trees is itself estimated based solely on the cocoa pods visible and counted in a photograph of the tree. The networks should therefore regularly process datasets from different cocoa tree plots.

Here we presented a comparative study of different neural network architectures for pod detection on colour images of cocoa trees. In particular, we compared Faster-RCNN [27], YOLOv5 [28], YOLOv8 [29], YOLOv11 [30], DETR-ResNet Vision Transformer [31] and the Real-Time Detection Transformer [32]. These networks were selected for three main reasons: (1) Faster RCNN is the original architecture that has historically been implemented for fruit detection, (2) the YOLO family has benefitted from continuous advances [33], and (3) transformer-based models are emerging as successors to CNNs [34]. The simplest and most complex architectures were deployed and the F1-score of each network was compared to assess the extent of precision loss, particularly in cases where the cocoa pod detector could be directly embedded in the smartphone or tablet acquiring the images. CNN ResNet50 and ResNet101 architectures [35] were compared with YOLO’s specific architectures. This study did not seek to investigate the optimal performance of each neural network trained and tested on a specific data set; it focused specifically on each trained network (performance) sensitivity to datasets. The study deliberately did not focus on the results of tests carried out during the network training sessions; rather, it focused on the results obtained on different validation datasets that were similar and representative of the data given to networks during early plot yield estimates. The study also aimed to assess which models for detecting cocoa pods could be installed on smartphones in order to eventually offer field solutions.

The first part of this article focuses on the data and their origins, the characteristics of the studied networks. The second part presents the values of the indicators used and the main results of the comparative study. Finally, the third part contains a general discussion on the results, their relevance and future prospects.

## Materials and methods

### Material

**Fig 1:**
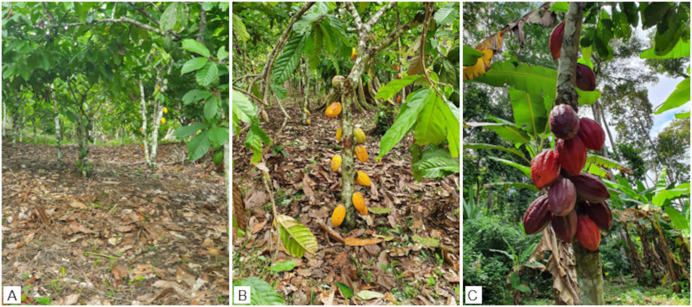
Forastero cocoa orchards, Abidjan district in western-central Côte d’Ivoire. The cocoa plots were natural plantations of relatively small trees with cocoa pods at various stages of development.

Cocoa (*Theobroma cacao*) is a small evergreen tree with sparse foliage belonging to the Sterculiaceae family. It produces edible cocoa beans with different flavours depending on the cocoa tree variety, and cocoa is the main ingredient in chocolate making [36]. These beans develop in the fruit (pods) of the cocoa tree. Cocoa pods are elongated and resemble a fairly rounded cucumber. They measure 15–25 cm in length and 6–15 cm in diameter, while weighing 300–500 g depending on the variety. These pods are usually reddish-yellow in colour when ripe (Fig 1). Because of the large mass of seeds contained in cocoa pods, the pod surface is covered with numerous small bumps, but also marked by around 10 relatively deep longitudinal grooves.

Cocoa growing is not mechanised in Côte d’Ivoire [37] and cocoa farms are fairly dense ‘natural’ orchards (Fig 1). Forastero is the most widely grown variety, which accounts for 79% of cocoa production in the country, whereas the Trinitario variety accounts for 20%. Criollo is the rarest cocoa variety, and is currently the least cultivated, accounting for just 1% of all harvested cocoa [38]. Our study focused exclusively on Forastero cocoa Image acquisition. A cocoa image dataset was acquired manually by an operator in cocoa crop fields in Côte d’Ivoire (July 2019). The images were captured using the main camera of a mobile Samsung Galaxy S10 SM-G973F phone in uncontrolled field conditions. No specific protocols were deliberately followed for image acquisition, and parameters such as shooting distances and focal lengths were not specified in advance. This choice was intentional and reflected real field conditions. Cocoa farms presented highly variable environments, particularly in terms of planting density, background, and fruit occlusion. Consequently, images in the dataset exhibited variations in depth of field and shooting angles. The lighting conditions and fruit occlusion were not controlled, and the images were taken without flash. Images of different random trees were captured, and the distance between the trees and the camera sensor was not consistently recorded. The dataset thus contained images acquired at less than one meter (Fig 1C), between one and five meters (Fig 1B), and more than 10 meters (Fig 1A) from the foreground tree. The absence of a controlled acquisition protocol and the use of a handheld device in variable real-field conditions produced a dataset for images with highly heterogeneous content. Such heterogeneity inevitably meant that the detection of cocoa pods insensitive to shooting conditions.

The resulting dataset consisted of 500 colour images with 4,600 x 3,456 pixel resolution. These images were then deliberately resized to a low resolution of 1,008 x 756 pixels in order to reduce processing times on both computer servers and smartphones.

Authorisation for the scientific use of the knowledge and data collected in the project was governed and guaranteed by the legal framework for European projects.

### Image annotation

Expert image annotation is very crucial as it enables the creation of datasets to train neural networks, and of validation datasets to assess their performance. This is a tedious and time-consuming task that often requires substantial resources, as the annotation quantity needs to be as high as possible to be able to conduct highly efficient performance studies. The authors deployed the cocoa-fruit-counting project on Zooniverse [39], the world’s largest participatory research platform [40]. Initially developed for the study of constellation images, the platform allows millions of volunteers around the world to be called upon to enhance research project resources, which was particularly helpful here for the image annotation stages.

**Fig 2:**
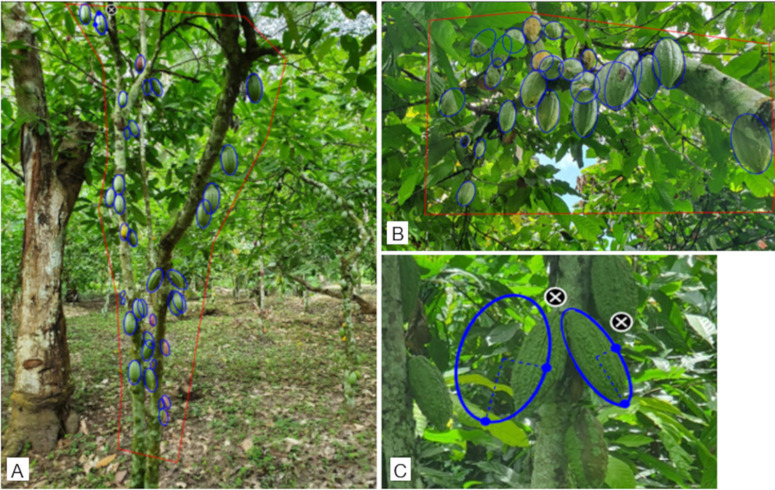
Zooniverse annotation. The foreground tree was outlined with a red polygonal line, and the cocoa pods were visible as blue ellipses.

The Zooniverse cocoa pod annotation process consisted of circumscribing each visible cocoa pod with a blue ellipse (Fig 2A, Fig 2B) that were manually positioned and adjusted (Fig 2C). As this research was part of a yield estimation study, each Zooniverse operator (trained expert) was asked to outline the foreground tree on each image with a red polygon (Fig 2ab).This simple polygonal line defined a mask of interest that did not allow for the exclusion of cocoa pods from the background trees visible through the foliage of the foreground tree. Furthermore, without very precise annotation guidelines, the assessment of the visibility of partially obscured cocoa pods or small cocoa pods was left to the discretion of the operators. Seven Zooniverse operators annotated each image in the dataset. Seven sets of similar but different annotations were thus associated with each image. These differences created a bias called annotation noise that needed to be removed. An indicator of overlapping bounding boxes around the ellipses made it possible to count the annotations common to each operator for each image, and thus the annotations not strictly common to each operator. Only 82% of the annotated cocoa pods were systematically annotated by all Zooniverse operators. In addition, there was more or less significant inter-operator variability in the size, orientation and position of the ellipses of the common annotations; no objective explanation could be found other than the interpretation of the guidelines or fatigue induced by the repetitiveness of the annotation tasks. To eliminate this annotation bias, we chose to represent each cocoa pod by the bounding box encompassing the fusion of its various annotations. That was the image truth.

### Training and validation datasets

**Fig 3:**
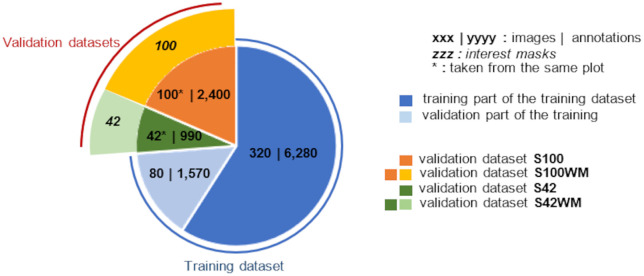
Training and validation datasets. The annotated images are divided into three non-overlapping datasets: the S400 training and the S42 and S100 validation datasets.

The annotated data was divided into disjoint training and validation datasets that did not share any data in common. The labelled annotations were deduced from the Zooniverse ellipses and consisted of rectangular bounding boxes with dimensions ranging from 10 to 100 pixels per side. These bounding boxes were drawn to as accurately as possible enclose the visible portions of the pods on the images. The training dataset included ~7,850 annotations representing diverse visual cocoa pod features in terms of shape, colour, sunlight conditions or occlusions on 400 images (S400) from different plots: the first 80% of annotated images were used to train the network, and the remaining 20% used to estimate the network’s performance during the various training loops. Two distinct unbreakable validation datasets were created with the remaining annotated data to assess variability in the trained networks with the aim of ensuring that the training and validation datasets would be representative of the overall data distribution. The first validation dataset (S42) consisted of 42 images that included 990 annotated pods. The second validation dataset (S100) contained 100 images with a total of 2,400 annotated pods. Each validation dataset consisted of images from a single plot, following the recommended data acquisition protocol for early yield estimation. The difference in dataset size is solely due to the difference in plot size. The plots were selected from the same geographical region, with the same cultivation practices and the same varieties of cocoa trees. The first plot was 3.5 hectares with a density of 1,200 trees per hectare, the second was 10 hectares with a density of 1,000 trees per hectare; these densities were in line with the African regional standard ARS 1000, which recommends between 800 and 1,600 trees per hectare for sustainable cocoa cultivation [41]. Each validation set corresponded to a random sample of 1% of the trees in each plot, in accordance with the minimum recommendations for estimating the yield of mango orchards [42]. Our study was part of the transposition of the mango protocol developed in Senegal for estimating the yield of cocoa plantations in Côte d’Ivoire. These two sets were therefore considered similar, the idea being to assess the sensitivity of the detection networks to the data. Furthermore, the polygons outlining the foreground tree were used to generate binary interest masks. These masks were black-and-white images on which the region defined by the polygonal boundary of the area of interest cocoa tree was rendered in white, while the remaining area was black. Added to datasets S42 and S100 respectively, they defined datasets S42WM and S100WM (Fig 3).

### Neuronal networks

The study was focused on Faster-R-CNN, YOLOv5, YOLOv8, YOLOv11, DETR-ResNet Vision Transformer and Baidu’s Real-Time-Detector networks. While the Region-based Convolutional Network (R-CNN), You Only Look Once (YOLO) and Transformers are object detection networks, their respective mechanisms differ. R-CNNs start by finding interesting parts of the image, then examine these parts more closely to determine what they contain. YOLO networks grid the space via successive convolutions and then locate and identify objects of interest. This model first emerged in 2015, but it was not until YOLOv5 (2021) was designed that the model became easily accessible and sufficiently optimised for acceptable detection. YOLOv8 (2023) was then the first model that offered truly significant features and improvements for enhanced detection performance, flexibility and efficiency. YOLOv11 (2024) is the latest iteration in the “classic” series of real-time object detectors. This version includes major improvements in the architecture and learning methods, so it is more versatile than its predecessors. Self-attentive Transformers models (2017) are conceptually based on the attention mechanism and take the context of the observations to be predicted into account. This type of model is particularly effective in translation tasks, where a sequence of words in one language is transformed into a sequence of different words in another language. Vision Transformers emerged in late 2020 and adapted the architecture of Transformers to process image content. These models are rapidly overtaking and replacing convolutional models for computer vision. The Real-Time Detection Transformer features significant optimisations for dealing with multi-scale features—this involved decoupling intra-scale interaction and inter-scale fusion, and making the model highly adaptable, thereby enabling flexible inference speed adjustment.

Yet beyond their differences, these networks share common features. They are all based on an arrangement of successive layers containing unitary computing elements called neurons. The neurons are all weighted and biased during training in order to be suitable for the detection of objects of interest. The total number of parameters is a linear combination of the weights, biases, inputs and outputs of each network layer. The Faster-R-CNN nano ResNet50 architecture (FRCNNn) has around 45 million parameters, and the Faster-R-CNN extra-large ResNet101 architecture (FRCNNx) around 55 million. The YOLOv5 nano architecture (YOLOv5n) has around 1.8 million parameters, and the YOLOv5 extra-large architecture (YOLOv5x) around 86 million. The YOLOv8 nano architecture (YOLOv8n) has around 3 million parameters, and the YOLOv8 extra-large architecture (YOLOv8x) around 86 million. The YOLOv11 nano architecture (YOLOv11n) has around 2.5 million parameters, and the YOLOv11 extra-large architecture (YOLOv11x) around 57 million. The DETR-ResNet Vision-Transformer nano ResNet50 architecture (ResNetDetrn) has around 26 million parameters, and the DETR-ResNet Vision-Tranformer extra-large ResNet101 architecture (ResNetDetrx) around 86 million. Finally, the Baidu’s Real-Time-Detector Large architecture (RTDetrl) has around 32 million parameters and the Baidu’s Real-Time-Detector extra-large architecture (RTDetrx) around 66 million. Furthermore, the networks can only structurally process square images of fairly low resolution between 300x300 and 1,096x1,096 pixels. When images are larger than the resolution expected by the network, the latter automatically resizes the native image, although this sometimes alters the image content.

### Image tiling

Small objects of interest may disappear or become indistinguishable after resizing the native images. To avoid this major inconvenience, it is advisable to divide the native image into thumbnails, or so-called tiles, of a resolution that can be processed by the networks, with small objects of interest remaining visible and detectable. A tiling strategy with tile overlap was adopted to ensure that each object of interest would be fully contained within at least one tile. The detection networks therefore provided bounding boxes locating cocoa pods in each tile. These bounding boxes were the network predictions. Post-processing was applied to transfer the cocoa pod detections obtained in each tile to the original image. This involved repositioning the predicted bounding boxes from the individual tiles on the coordinate system of the full image. Due to tile overlapping, duplicate detections of the same cocoa pod could occur across adjacent tiles, so a process was implemented to delete duplicate predictions.

### Hyperparameter tuning

Hyperparameter tuning is not just a one-off configuration, but an iterative process aimed at optimising the performance metrics of the machine learning model, such as accuracy, precision and recall. These hyperparameters are numerous – around 30–40 depending on the model – and varied. They range from learning rates to architectural details, such as the number of layers or the types of activation functions used, to the specification of data enrichment functions. Optimising them is not easy, despite the existence of known techniques such as practical swarm optimisation, Bayesian optimisation algorithms, and genetic algorithms [43]. For this study, the hyperparameters were set by default according to the recommendations of recent studies comparing the performance of fruit identification networks, particularly YOLO and Faster RCNN [44], Real-Time Detector [45] and Vision Transformers [46]. Tuning was deliberately limited to the following major hyperparameters: first, the learning rate, which determines the size of the model parameter update step during optimisation, influences the speed and stability of the convergence process and therefore effectively minimises the loss function; then the momentum, traditionally used to accelerate the descent of the gradient in the right direction, which allows learning to converge more quickly; finally, the weight decay parameter, which prevents overfitting by penalising large weights, ensuring that the model generalises well to new data sets. The most difficult parameter to define was the batch size: this parameter depends heavily on the model architecture, the possible number of samples to be processed before updating the model’s internal parameters, and the available hardware resources. There is no universal rule for determining the best batch size. As a result, we have chosen to set it identically for all networks, which undoubtedly penalised some of them. It was therefore set at 8 for all networks, as this was the only possible value for the largest architecture studied in view of the available resources. In addition, complementary parameters were set identically for the different networks: for example, the data augmentation parameters that help the model become invariant to object direction or colour variations. Finally, parameters specific to each network (families) were customised: for example, the epoch, which represents a complete pass through the entire data set; the iteration, which represents the passage of a batch of data; and the patience, which indicates the number of epochs to wait without improvement in validation metrics before stopping training early. A learning rate of 0.1 was taken from the aforementioned articles for YOLOv5, YOLOv8, YOLOv11 and Baidu’s Real-Time Detector, 0.001 for Faster R-CNN, and 0.005 for Vision Transformer. Momentum was set at 0.937 for YOLO and 0.9 for the others networks. The weight decay parameter was set to 0.0005. The dataset was systematically augmented by right-left flip and slight variation in the hue and saturation of the initial data. Systematic 90° rotations of the dataset were applied to ‘show’ horizontal cocoa pods, which occur when images accidentally switch to landscape mode due to the orientation of smartphones but are processed in portrait mode.

The maximum number of epochs was arbitrarily set at 1,000 with a patience of 100 for YOLO and Baidu’s Real-Time Detector. The 400 images in the training set were divided into 1,600 tiles, or 200 batches of 8 tiles; the maximum number of iterations was therefore set at 200,000 for the Faster RCNN and Vision Transformer (Table 1).

**Table 1:** Network hyperparameter tuning.

Hyperparameters	YOLOv5, v8 & v11 Baidu’s RTDetr	FRCNN	ResNetDetr
learning rate	0.01	0.001	0.005
batch size	8	8	8
momentum	0.937	0.9	0.9
weigth decay	0.0005	0.0005	0.0005
image sizes	640	640	640
number of iteration	–	200,000	200,000
number of epochs	1,000	–	–
patience	100	–	–
hue augmentation	0.03	–	–
saturation augmentation	0.3	–	–
hozizontal flip	1 (systematic)	random	random
rotate	90°	90°	90°

### Design of experiments (DOE)

Each network was trained on the S400 training dataset and its performance was evaluated on the two disjoint validation datasets S42 and S100, with or without interest masks. The validation of datasets with interest masks consisted of pairing only expert annotations and network predictions whose geometric centre of the bounding box belonged to the area of interest. Benchmark performances were provided based on the S400 training set. The hyperparameters for each network were left at the default values as described in Table 1.

The normalized rate of geometric overlap between two bounding boxes was used to measure the total or partial superposition of objects. The intersection on union (IoU) of objects was defined by the ratio of the intersection to the union of the two bounding boxes [47]. This latter indicator was applied for the deletion of cocoa pods predicted several times by the networks due to tile overlap bands and for matching expert annotations with network predictions. The value of this ratio was compared with a pre-determined threshold and used to identify overlapping boxes considered to represent the same cocoa pod. We chose to evaluate the detection performance of the networks on different validation sets using the F1-score [48]. This was the most well-known indicator and the one most used by the application community. The F1-score was the harmonic mean between the precision and recall, two statistical indicators giving the contribution of ‘false positives’ and ‘false negatives’ respectively to the network’s overall detection error. The true positives were cocoa pods annotated by the expert and correctly detected by the network. The false negatives were cocoa pods annotated by the expert but not detected by the network. The false positives were also cocoa pods detected by the network but not considered as such by the expert. False negatives and false positives are classic network errors that we generally seek to reduce.

The performance indicators were assessed by cocoa pod size class. Cocoa pods framed by boxes smaller than 16x16 pixels were considered ‘small’, cocoa pods framed by boxes between 16x16 and 32x32 pixels were considered ‘medium’, and cocoa pods that did not fit into either of the above categories were considered ‘large’.

All the experiments were carried out on the AgroDeep platform [49], thereby ensuring that the technical environments were relatively similar between the different tests. Python scripts for pre-processing the data, post-processing the results and inferring the neural networks were encapsulated in Singularity containers [50], which provided an optimal operating environment (operating system, additional calculation packages, CUDA drivers, etc.) for each network in the study. All training and validation procedures were carried out on the same computing server, which was exclusively dedicated to the study, and equipped with a 24GB Nvidia Quadro RTX6000 graphics card, 4,600 CUDA cores and 570 Tensor cores.

The tile size was set at 640x640 without overlap for training all the networks and at 640x640 with a 200 pixel overlap for validating all the networks. The threshold for removing multiple predictions was set at 0.25. A minimum IoU threshold of 0.7 was set to determine a successful match between the network predictions and the expert ground truth annotations.

## Results

The initial results provided a better understanding of the main limitations of the study.

### Network training performance

**Fig 4:**
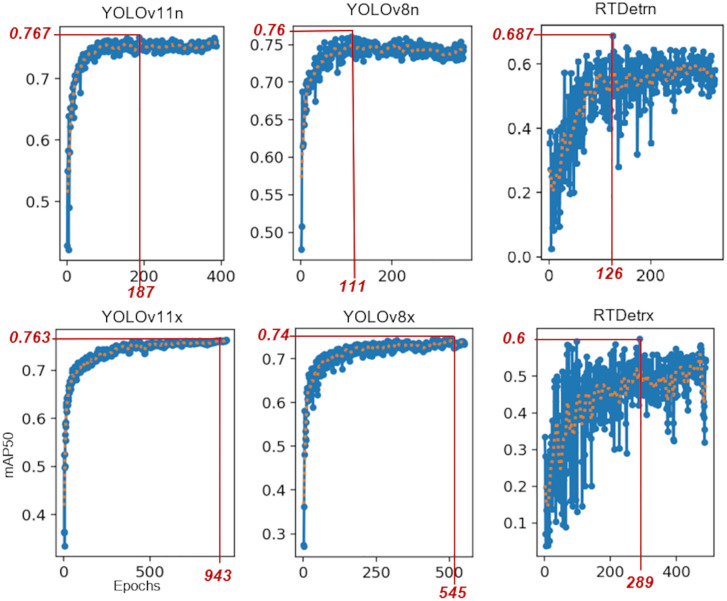
The mAP50 training curves of the nano and extra-large architectures of the YOLOv8, YOLOv11, and RTDetr networks.

The mAP50 performance metrics measured the average precision at the IoU threshold of 0.5 alone. This metric verified whether the model could correctly find objects with a fairly flexible accuracy requirement. It focused on whether the cocoa pod was roughly in the right place, without requiring perfect placement, and was therefore representative of the detection of cocoa pods alone. The mAP50 evolution curves over the course of training for nano and extra-large architectures showed the limitations encountered by the YOLOv8, YOLOv11, and RTDetr networks (Fig 4). These performance curves were quite similar in shape and amplitude from one network to another, including networks not shown in the figure. The optimal mAP50 values were added in red on the y-axis and their corresponding epochs on the x-axis. No values higher than 0.8 for nano and extra-large architectures. With a maximal mAP50 of 0.763 in the 943rd epoch for its extra-large architecture and 0.767 in the 187th epoch for its nano architecture, YOLOv11 gave the highest precision. The median accuracy represented in orange on the mAP50 curves was 75%. YOLOv8 showed a slightly lower maximum mAP50 of 0.7 for its extra-large architecture and 0.6 for its nano architecture. RTDetr fell to 0.6 and 0.7 respectively, showing a slight decline. The performance curves of YOLOv5, Faster RCNN and Vision Transformer was significantly lower and were therefore not shown in Fig 4. Regardless of the networks, the extra-large architectures unsurprisingly required 2–3 times more epochs than the nano architectures to adjust the weights of their parameters, which were 10–20 times more numerous than those of the nano architectures. The constant oscillation of the performance curves was normal for YOLO but a little excessive for the other models; these differences in oscillation amplitudes were explained by the different learning rate settings.

### Validation of trained networks

The performance of the trained networks was evaluated both at the level of the entire image and at the level of the foreground trees alone. In the first case, the entire image was considered, and all predictions for each image were taken into account. In the second case, only predictions falling within the masks of interest defined for each image were taken into account. This allowed for a detailed analysis of detection accuracy, both in general and in specific regions.

**Fig 5:**
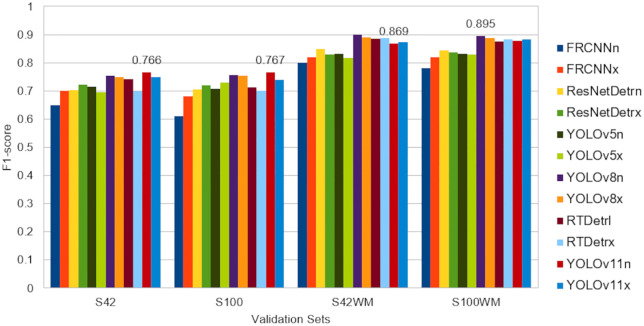
Summary of the performance of different networks. Network performance was estimated using the F1-score for each validation datasets. The results were ranked from oldest to newest network.

The S42 and S100 datasets illustrated the network performances at the entire image scale, while the S42WM and S100WM datasets illustrated these performances solely at the foreground tree scale. Unsurprisingly, the most recent networks (published as of 2023) gave better results than the older networks (Fig 5). The top three were clearly RTDetr, YOLOv8 and YOLOv11. YOLOv11n achieved an F1-score of ~77% for theS and S100 datasets. The YOLOv8 and RTDetr networks, regardless of their respective architectures, ranked just behind with F1-scores ranging from 75.3% to 75.7%. The trend was, however, reversed for the S42WM and S100WM datasets: with an F1-score of ~90%, YOLOv8n was around 2% better than YOLOv11 and RTDetr. With a maximum difference of 3%, the F1-scores of the nano and extra-large architectures of the YOLOv8 and YOLOv11 networks were very close, and even equivalent. They were substantially higher than those of the RCNN, YOLOv5 and transformers, with a difference of >5%, all architectures combined. Pre-experimentation on the validation of the different networks trained on a set of 255 images containing 4,527 annotated cocoa pods led to a systematic performance decrease of 5–15% on the S42, S100, S42WM and S100WM datasets.

The histograms for S42 and S100 (resp. S42 WM and S100WM) were similar in shape and value (Fig 5). This similarity in shape and amplitude indicated that the networks were relatively insensitive to the size of the validation data sets, provided that these were representative of the same reality.

Assessing the sensitivity of network performance to datasets sizes was difficult with so little data available. However, trends were already emerging when comparing network performance obtained on the S42 and S100 validation datasets, first without and then with the use of interest masks (Fig 5). The largest variation observed was 4% for FRCNN; this variation fell below 1% for ResNetDetr (n&x) r, YOLOv5 (n&x) and RTDetrl, and fell to less than 0.5% for the others. The candidate networks were therefore those that performed close to 90% with a variation of less than or equal to 0.5%.

We therefore focused our study on the top three networks with high performance on validation datasets and low performance variations between validation datasets.

### Performance of trained networks by cocoa pod size class

Colour coding of the cells ranging from red for the lowest scores to green for the highest scores.

The colouring of the tables quickly showed that the networks had more difficulty in detecting small background fruit than large foreground fruit. The penultimate row of the table gave the average scores per fruit size class for cocoa pod detection on whole images, while the last row showed the cocoa pod detection scores only in interest masks. A slight improvement in the average F1-scores per network was observed when the cocoa pod detections were limited to the foreground trees.

The study of performance by image size class of cocoa pods provided a better understanding of the strengths and weaknesses of the trained networks. With F1-scores not exceeding 50%, the networks demonstrated their poor ability to detect small fruits whose bounding box size was less than 16x16 pixels (Table 2). A F1-score of 30% corresponded to approximately four-fifths detection errors, and a F1-score of 50% to approximately two-thirds detection errors. This category of fruit included both foreground cocoa pods at an embryonic stage of development and background cocoa pods at an intermediate stage of development. For most of the networks evaluated, the F1-score was significantly increased by restricting performance to the foreground tree alone: it went from 38.8 to 50.1 for YOLOv8n, from 40.3 to 51.8 for YOLOv8x, etc. This increase in performance reflected the partial exclusion of background fruits through the use of masks of interest. RTDetrl showed a decline in performance, suggesting a lack of network training. Performance rose above 80% for medium-sized cocoa pods with bounding box dimensions between 16x16 and 32x32 pixels (Table 3). An F1 score of 80% corresponded to approximately one-third detection errors. This category of fruit contained both cocoa pods in the foreground at an intermediate stage of development and pods in the background at a mature stage of development. The F1 score was significantly increased by restricting performance to the foreground tree alone: it went from 84.6 to 88.7 for YOLOv8n, from 84.5 to 88.4 for YOLOv8x, etc. The networks therefore performed acceptably, although there was room for improvement for this category of fruit size. Performance rose above 92% for large cocoa pods whose bounding box dimensions were greater than 32x32 pixels (Table 4). An F1-score of 90% corresponded to approximately one-sixth detection errors. This fruit category contained large cocoa pods in the foreground at a mature stage of development, either fully visible or partially obscured. The F1-score was slightly higher when performance was restricted to the foreground tree alone: it went from 95 to 95.5 for YOLOv8n, and from 93.9 to 94.5 for YOLOv11n, etc. The networks therefore performed adequately for this fruit size category. The difficulties encountered were probably related to the degree of fruit occlusion.

**Table 2:** F1-Scores of nano and extra-large architectures of YOLOv8, YOLOv11, and RTDetr networks for small cocoa pods.

%	YOLOv8n	YOLOv8x	YOLOv11n	YOLOv11x	RTDetrl	RTDetrx
S42	31.7	31.6	33.9	35.7	37.6	35.5
S100	45.5	49	48.6	39.3	42.9	43.6
S42WM.	54.5	55.7	52	46.6	25	37.2
S100WM	45.7	47.9	42.4	41.3	30.9	41.1
***av. NWM***	***38.6***	***40.3***	***41.3***	***37.5***	***40.3***	***39.6***
***av. WM***	***50.1***	***51.8***	***47.2***	***44***	***28***	***39.2***

**Table 3:** F1-Scores of nano and extra-large architectures of YOLOv8, YOLOv11, and RTDetr networks for medium cocoa pods.

%	YOLOv8n	YOLOv8x	YOLOv11n	YOLOv11x	RTDetrl	RTDetrx
S42	85.6	85.8	80.9	84.6	81	81.9
S100	83.5	83.2	83.6	83.7	77.7	80.4
S42WM.	89	88.6	85.2	87.9	86.2	86.9
S100WM	88.4	88.1	86.3	88.4	85.3	87.2
***av. NWM***	***84.6***	***84.5***	***82.3***	***84.2***	***79.4***	***81.2***
***av. WM***	***88.7***	***88.4***	***85.8***	***88.2***	***85.8***	***87.1***

**Table 4:** F1-Scores of nano and extra-large architectures of YOLOv8, YOLOv11, and RTDetr networks for large cocoa pods.

%	YOLOv8n	YOLOv8x	YOLOv11n	YOLOv11x	RTDetrl	RTDetrx
S42	94.9	95.1	93	91	91.4	93.3
S100	95	94.5	94.8	93.3	92	94.2
S42WM.	95.3	93.8	93.6	92	94.3	94.1
S100WM	95.7	95.3	95.3	94	94.1	94.1
***av. NWM***	***95***	***94.8***	***93.9***	***92.2***	***91.7***	***93.8***
***av. WM***	***95.5***	***94.6***	***94.5***	***93***	***94.2***	***94.1***

The networks could be ranked according to their respective performances solely based the analysis of the results obtained for the trees of interest. An average F1-score of 43% for small cocoa pod detection, but with a dispersion of ~9%, showed that the RTDetr architectures did not perform well in this size range. An F1-score of 87% for medium-sized pod detection with a dispersion of <1.5% illustrated the similarity of the responses of the three networks, although YOLOv8 had a slight advantage over YOLOv11 and RTDetr. With an F1-score of 94% for large cocoa pod detection with a dispersion of <1%, YOLOv8, YOLOv11 and RTDetr confirmed their ability to identify and count pods on foreground trees. With average F1-scores of 78% across all size classes, the extra-large and nano architectures of the YOLOv8 network were ~3% better than YOLOv11 and ~6% better than RTDetr. But with an F1-score of 95.5% for the large fruit class alone, the YOLOv8 nano architecture performed >1% better than all the other architectures.

### Matching expert annotations and network detections

A qualitative assessment was achieved by images showing the match between expert annotations and network predictions: the true positives were represented by blue bounding boxes, the false negatives by yellow bounding boxes and the false positives by pink bounding boxes in the zones of interest on the foreground tree. Although not easy to appraise, the blue and pink boxes showed the location of the network predictions, which meant that there could potentially have been slight differences between networks.

**Fig 6:**
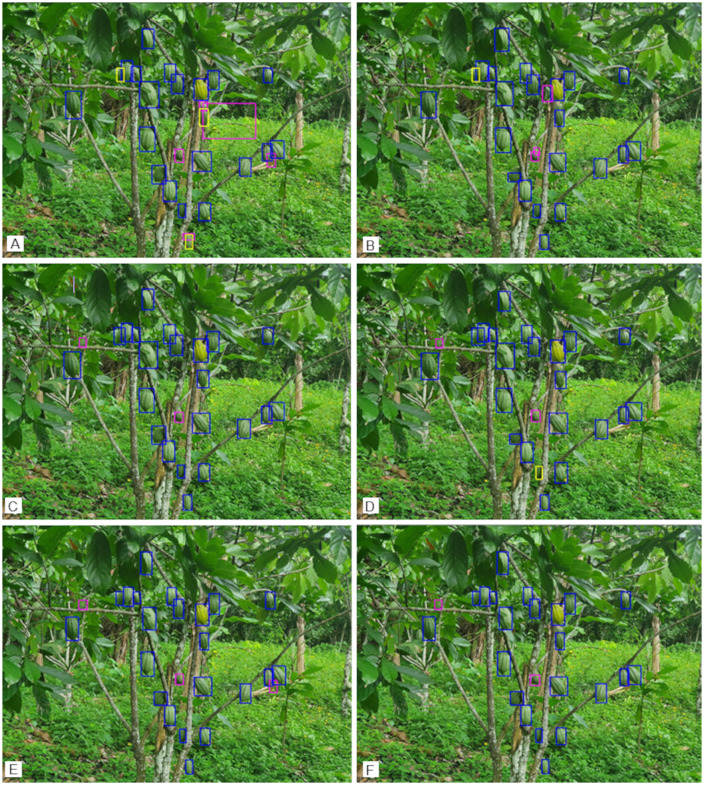
Qualitative visual assessment of prediction accuracy for YOLOv11 (top), YOLOv8 (middle) and RTDetr (bottom) in nano (left) and extra-large (right) architectures. The prediction boxes are coloured according to the annotation/prediction appearing classes: true positives in blue, false negatives in yellow, and false positives in pink.

On the foreground tree, YOLOv8n (Fig 6C) and RTDetrx (Fig 6F) obtained the fewest errors, with 22 true positives and two false positives, for a local F1-score of 95.6%. All annotated cocoa pods were detected, and also the network detected two false positives. The pink box on the left was probably a leaf whose shape and orientation misled the network, even though there was still some uncertainty about its interpretation. However, the pink box in the centre is a cocoa pod that was not annotated in the ground truth. The network was therefore penalised for detecting a cocoa pod that the human expert had not seen and annotated. Adding this box to the expert annotations of the ground truth would have allowed it to be considered as true positive, thereby increasing the local F1-score to 97.8%.

Paradoxically, YOLOv8x (Fig 6D) also missed a cocoa pod, despite having a more complex architecture than YOLOv8n. The YOLOv11x (Fig 6B) seemed to be more efficient than YOLOv11n (Fig 6A), which generated numerous detection errors, although not as efficient as the YOLOv8. With three false positives and a local F1-score of 93.3%, RTDetrn (Fig 6F) ranked equally with networks presenting one false negative and two false positives (Fig 6B, Fig 6D), even the absence of false negatives was a good thing.

### Matching annotations/detections and heterogeneous data

**Fig 7:**
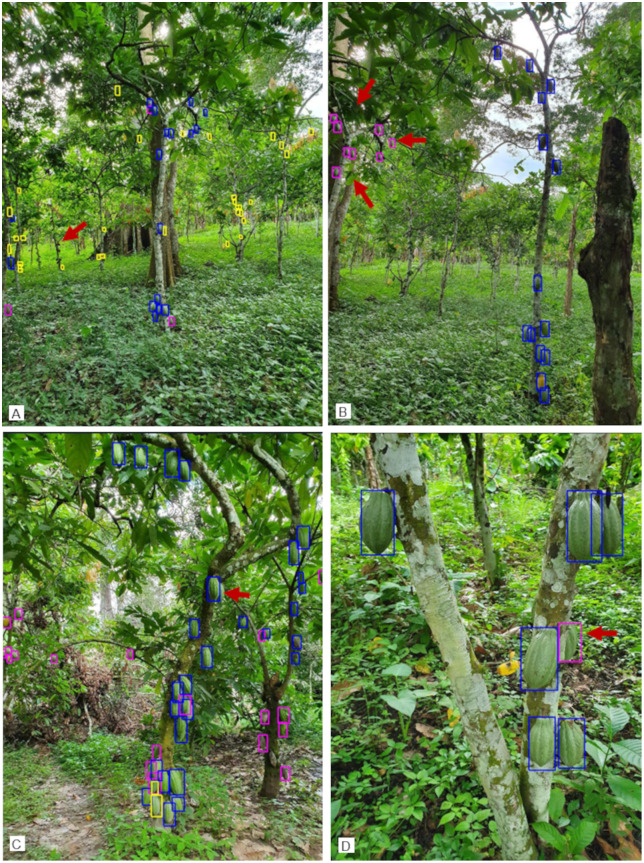
Impact of heterogeneity in acquisition and annotation practices on the performance of the YOLOv8 nano network. The prediction boxes are coloured according to the annotation/prediction appearing classes: true positives in blue, false negatives in yellow, and false positives in pink.

As previously mentioned, without a strict acquisition protocol, the cocoa pods to be detected displayed a significant variation in terms of geometric and textural characteristics from one image to another (Fig 7). Furthermore, the use of Zooniverse annotators clearly accentuated the heterogeneity of the image annotations. Case of a foreground tree located at a distance of 10–12 meters (Fig 7A): the false negatives in yellow showed a tendency to annotate all the cocoa pods on the background trees, even though some of them were ignored (see the red arrow). The height of the larger cocoa pod was less than 10 pixels. The network was unable to detect these small fruits. The image displayed a local F1-score of 50%, and the foreground tree of 83%. Case of a foreground tree in a regular plantation located at a distance of 1–2 meters (Fig 7.B): the cocoa pods on the background trees were not annotated, even though the height of the smallest fruit was greater than 10 pixels.: however, background fruits with a height greater than 15 pixels were detected by the network and became false positives shown in pink (see red arrows): the network was penalized for correctly detecting cocoa pods that were intentionally or unintentionally ignored by the human expert. The image displayed a local F1-score of 77.7%, and the foreground tree of 100%. Case of a foreground tree in a free plantation located at a distance of 1–2 meters (Fig 7.C): the network found itself in the same situation as before; note the half-hidden cocoa pod that was neither annotated by the human expert nor detected by the network: a normal anomaly in a way. The image displayed a local F1-score of 72%, the foreground tree of 85.7%. Case of a foreground tree located at a distance of less than one meter (Fig 7.D): here, the height of the cocoa pods was approximately 200 pixels. The network detected all annotated pods but was penalized for detecting a fruit partially obscured by the trunk, which was intentionally or unintentionally ignored by the human expert. The image and the foreground tree displayed a local F1-score of 92.3%. The only constant in all these examples was that the network performed fairly well on the foreground tree.

### CPU and GPU times

The CPU and GPU runtimes for training and network validation were defined by differences between the system times taken at the start and end of each key stage. The server used was exclusively dedicated to the calculations so as to ensure inter-network comparability of calculation times. Input data tiling, removal of multiple predictions, pairing of expert annotations and network predictions and processing performance estimations were carried out via the computer’s CPU, whereas the learning and prediction of networks were carried out via the computer’s GPU. The CPU time to load data from the hard disk to the graphics card memory during GPU processing was overlooked.

**Fig 8:**
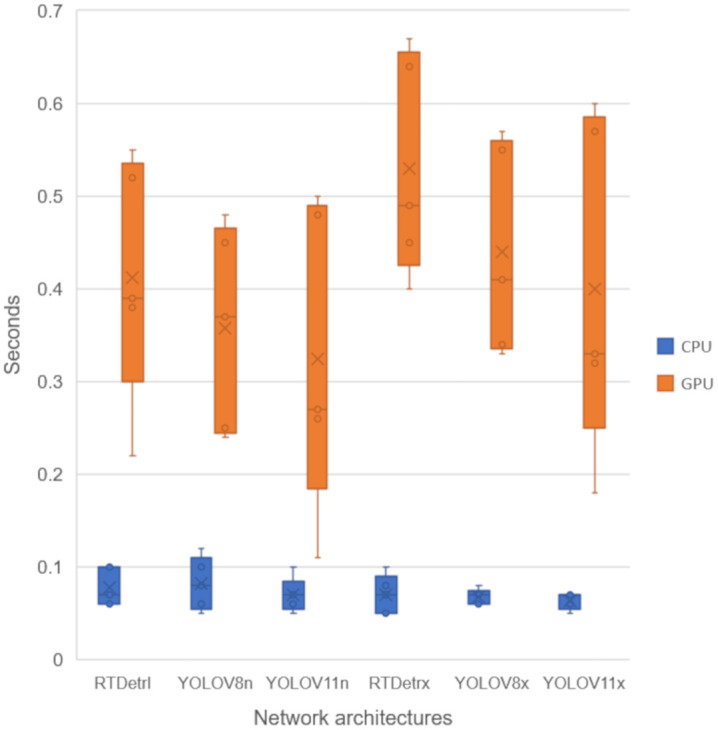
Mean CPU (central processing unit) and GPU (graphical processing unit) times for RTDetr, YOLO8 and YOLO11 validations per image. Average times were assessed solely on the S100 and S42 validation datasets.

The average unit times per type of IT resource were evaluated across the S100, S42, S100WM, and S42WM validation datasets. The boxplots (Fig 8) summarise the datasets at six key figures, i.e., the minimum (lower horizontal line), the 25th percentile (lower circle), the median (horizontal line), the mean (cross), the 75th percentile (upper circle) and the maximum values (upper horizontal line), thereby facilitating the detection of outliers in the distribution of resource consumption values, which could indicate instances of particularly high or low resource usage. With a tiny dispersion of 0.0066 s, the average CPU times/image between the different networks were considered to be similar—a very logical result given that the input data pre-processing and output post-processing algorithms were rigorously identical. With a significant dispersion of 0.071 s, the GPU times illustrated the impact of the different complexities and mechanisms of the architectures tested. RTDetr transformer was clearly the slowest network, with an average GPU time/image of 0.41 s for its simplest architecture and 0.53 for its most complex architecture. YOLOv8 was the fastest network, with an average time of 0.32 s for its simplest architecture and 0.40 s for its most complex architecture. However, the YOLOV8n boxplots showed outlier distributions with a median value tending towards the 25th percentile unlike YOLOv8xwhose median value tended towards the mean.

## Discussion

With overall F1-scores <80% on all images and barely 90% on foreground trees, cocoa pod detection was less successful than the detection of apples and sweet peppers [11], mangoes [12], oranges [14] and lemons [19], which had F1-scores of 92–98%. This poor performance needed to be explained.

Without an imposed acquisition protocol, the shooting distances to the foreground tree varied from less than 1 meter to more than 10 meters, as shown in Figs 1 and 7. The widening of the shooting point field increased the complexity and detail of the background. The images contained cocoa pods of varying sizes, ranging in height from less than 10 pixels to more than 200 pixels; some of them were in shadow, others were moderately lit or overexposed; finally, they clearly showed different stages of development, as illustrated in Fig 1. The set of images was therefore highly heterogeneous, reflecting the reality on the ground; however, it did not meet the requirements for detecting fruit only on foreground trees, which was the important point of a study aimed at estimating yield. In this specific case, excessive heterogeneity resulted in overly varied fruit configurations, most of which did not apply to the foreground trees; this heterogeneity proved to be potentially counterproductive.

The Zooniverse expert annotations undeniably produced an inconsistent annotated dataset with numerous biases. Fig 7 showed annotation errors or inconsistencies, estimated at around 40%. Because it was difficult to control the profiles of Zooniverse operators, it was impossible to guarantee the quality and consistency of the annotation work. But the real problem stemmed from the excessive heterogeneity of the data, which ultimately left too many different interpretations of the annotation guidelines. “Annotating visible fruits” was a problem of definition concerning the size and partial occlusion of cocoa pods, which had to be taken into account or ignored. An inconsistent annotated dataset penalized the networks in two ways. First, it made the network learning process complex and slow by systematically showing them examples and counterexamples of learning; the learning examples were all cocoa pods explicitly annotated by the Zooniverse operator, particularly in the background contexts; the learning counterexamples were all fruits resembling the learning examples but intentionally or unintentionally not annotated by the Zooniverse operators. In the presence of numerous counterexamples, the networks struggled to understand and reproduce the operators’ expectations, which resulted in particularly slow convergence speeds, as illustrated in Fig 4. YOLO thus required two to three times more epochs than the standards displayed in most studies, for a rather average result. Second, it penalized networks that correctly detected cocoa pods that had not been annotated by Zooniverse experts. Fig 5 showed that limiting the detection of cocoa pods to trees in the foreground significantly improved performance, from 75% to 90%. The foreground tree contained fewer small fruits and was therefore less prone to detection errors. However, as previously noted, a simple outline could be used to define the foreground tree but not to exclude fruits from background trees visible through the foreground tree.

The relationship between the size of the training dataset and network performance has long been known [51]. The 11,240 annotations were normally sufficient to conduct a relevant study. However, this did not take into account the inconsistency of the annotated dataset. Table 2 showed the difficulty networks had in maintaining consistent performance depending on the size class or location of the cocoa pods. The networks were twice as effective at detecting large cocoa pods as small ones, regardless of architecture and validation sets: performance reached 95% for the detection of large fruits compared to 45% for small ones; detection performance for small fruits was increased by 5–10% when limited to trees in the foreground, and by about 2% for large fruits. These results illustrated the impact and location of Zooniverse annotation biases.

In the absence of a precise annotation protocol, reducing network performance to only large pods could be a good alternative, since the networks are quite effective with F1-scores of around 94%. However, the approach of expressing fruit size in pixels was not particularly relevant. It could be improved by switching from pixel dimensions to metric dimensions, which is not so simple with poorly controlled sensors. Pixel sizes refer to the image and not to the physical size of the fruit: a large fruit on a background tree is small in the image.

The cocoa pod detection networks were ranked according to two complementary criteria: the value of their respective performance and the low sensitivity to the data of this performance. Direct interpretation of the inter-network classification results was therefore complex. Score differences >5% were considered relevant enough to validate the inter-network ranking, but differences <5% were not considered sufficiently relevant: we considered that these differences could be explained more by the datasets used than by the actual network capabilities. In this specific case, the performance of the networks could be considered equivalent, and the final choice of model to be implemented in an operational solution could be based on an additional criterion such as processing time. Beyond this classification process, our study aimed to assess whether the decline in performance of architectures that favoured speed over accuracy was still acceptable for counting cocoa pods. Against all expectations, YOLOv8n and YOLOv11n achieved F1-scores that were a few hundredths higher than those of the extra-large architectures.

With mAP50 values below 80% (Fig 4), the networks could be considered under-trained and required further tuning of their hyperparameters. However, with large cocoa pod detection performance ranging from 91% to 96% depending on the networks and datasets (Table 2), the trained networks showed good ability to process the foreground trees that were the target of interest in the study. Performances close to 90% were thus obtained by the YOLOv8, YOLOv11 and Baidu’s RTDetr networks on the only foreground trees (Fig 5). With such results, it was not necessary to re-tune the network hyperparameters but rather to correct the annotated or interest masks in the datasets.

However, with an optimum mAP50 consistently lower than that of the nano architectures (Fig 4), the extra-large architecture networks seemed to indicate a training defect. Normally, extra-large architectures should perform significantly better than nano architectures. The most likely explanation was that the training dataset was not rich enough to fully train the large architectures. The extra-large architectures had 20- to 30-times more parameters to weight than the nano architectures. Indeed, they would have needed much more data to achieve training of equivalent quality [52], which would be necessary to be able to truly objectively compare the networks.

The performance sensitivity study was difficult to conclude due to the obvious lack of consistent data. However, the two validation datasets provided initial trends that will need to be verified on a larger validation dataset without annotation noise. If these trends were confirmed, they would enable us to define a few rules for selecting candidate neural networks. It is not unreasonable to exclude from the list those networks whose performance variations exceeded 0.5%.

The CPU times per image were stable, even though inter-network variations of <0.007 s were observed. These variations did not markedly influence the time required to count cocoa pods in a plot yield estimation workflow. Otherwise, a marked 0.007 s fluctuation in GPU time was noted. This could undoubtedly be explained by the different complexities of the assessed neural architectures: YOLOv11n had 2 million parameters, while YOLOv8x had nearly 68 million. GPU times for cocoa pod detection would therefore have an impact on the time to count cocoa pods in a plot yield estimation workflow. Yet the GPU time seemed to be an interesting criterion that could potentially be used to complement the network classification. RTDetr networks were discarded because their average GPU times per image were 15–20% higher than those of YOLOv8 and YOLOv11, even though their average cocoa pod detection capacity was only 2% lower than that of YOLOv8.

The persistent and temporary data storage sizes were easy to evaluate for the different neural architectures when the algorithmic implementations were perfectly mastered. For example, the weight file size was constant for a given neural architecture, while ranging from a few kilobytes to several megabytes depending on the case.

For future work, it would be essential to make the datasets more consistent by removing annotation inconsistencies. These corrections could be made using the IJ-checker and IJ-annotator plugins [53]. The first allows to visually check false positives and switch them to expert annotations when they are actually fruits; the second allows to review and complete expert annotations, image by image. It would also be necessary to define a data acquisition protocol that focuses more on the foreground tree in order to limit the profusion of small cocoa pods. Size filtering could be applied when tiling the data, both for learning and validation. Increasing the size of the dataset would also be an interesting direction to pursue. All of this would likely significantly improve the performance and robustness of the networks to confirm the results of this initial study and produce more meaningful statistical studies on processing times. The performance of networks by cocoa pod size class must be investigated in future work. A calibration target, such as a blue sphere of known diameter, could be introduced to calibrate the image. For this additional study, new annotated images would be required in which a blue sphere is suspended at the edge of the foreground tree. A two-class network could detect and identify the blue sphere and the various visible cocoa pods. The bounding box of the sphere would allow the image to be calibrated and thus approximate the size of each pod, thereby refining the study of performance by size class. The trends noted in this first comparative study will now have to be confirmed in further research, particularly regarding the predominance of the YOLOv8 nano architecture. If these trends are confirmed, the real performance of this latter architecture will have to be assessed with an embedded system so as to obtain all of the elements needed to define the technical foundations of an operational solution to help estimate the yield of cocoa tree plots. However, network performance and speed are probably not the only criteria to consider when defining future solutions. Recent studies have shown that Snapdragon processors used in smartphones incorporate NPUs (Neural Processing Units) dedicated to AI and machine learning tasks. Mobile applications implementing a complete pipeline for data detection and classification with very low energy costs were already operational and opening up new application prospects [54]. Carbon footprint could thus become a major criterion in the development of business solutions.

It would thus be essential to enrich the datasets with truly new annotations representative of the different field contexts. This would significantly improve the performance and robustness of the network, while increasing the amount of data to confirm the results drawn from this study and enable more meaningful statistical studies on processing times to be conducted. A calibration pattern, e.g., a blue sphere of known diameter, could be introduced to enable image calibration, which in turn would enhance the precision on cocoa pod distributions by size class. The trends noted in this first comparative study will now have to be confirmed in further research, particularly regarding the predominance of YOLOv8n. If these trends are confirmed, the real performance of this latter architecture will have to be assessed with an embedded system so as to obtain all of the elements needed to define the technical foundations of an operational solution to help estimate the yield of cocoa tree plots.

## Conclusion

In this study, the lightest and heaviest architectures of the Faster R-CNN, Detr-ResNet Vision Transformer, Baidu’s Real Time Detection Transformer, YOLOv5, YOLOv8, and YOLOv11, with identical hyperparameters, were trained with 7,850 annotated pods from 400 low-resolution images of different cocoa plots. Each network architecture was validated on two complementary datasets that had not been involved in the training phases. Two validation datasets were defined in accordance with the recommended data acquisition protocol for early yield estimation, each corresponding to a cocoa plot. The S42 validation dataset comprised 42 images containing 990 annotated cocoa pods, and the S100 dataset comprised 100 images containing a total of 2,400 annotated cocoa pods. The validations were based on two modalities: detection of cocoa pods in the entire set of images, and otherwise in the foreground tree circumscribed by a specific polygonal line.

Unsurprisingly, the most recent networks published as of 2023 gave better results than the older networks. The top three networks were clearly RTDetr, YOLOv8 and YOLOv11. With average F1-scores close to 48% for detecting cocoa pods in the background, 88% for those in the middle, and 94.5% for those in the foreground, all with a maximum dispersion of 2%, the study confirmed that the YOLOv8, YOLOv11 and RTDetr networks were good candidates for identifying and counting cocoa pods on foreground trees with sufficient accuracy. However, with an F1-score of ~77% for the detection of cocoa pods in all images and 90% for foreground cocoa trees, YOLOv8n outperformed the other two by almost 3%.

The poor training performance of networks capped at 80% pointed to problems with hyperparameter tunings or dataset compositions. A summary study of annotation/detection pairings highlighted the inconsistency of the annotated dataset, which showed numerous annotation noise, particularly in the background of the images. The lack of acquisition protocols increased the heterogeneity of the data, causing most of the problems that explained the poor performance in both training and validation of all networks, regardless of their different architectures.

Beyond this ranking, our study was designed to assess whether the drop in the performance of nano architectures—which, unlike the extra-large architectures, prioritized speed to the detriment of precision—was still acceptable for yield estimates. Against all expectations, YOLOv8n and YOLOv11n achieved F1-scores that were a few hundredths higher than those of the extra-large YOLO and RTDetr architectures. Consequently, the extra-large architectures—with 20- to 30-times more parameters to adjust than the nano architectures—would probably have required larger datasets to be able to set up truly relevant network training sessions so as to enable fully objective inter-network comparisons.

However, one of the major aspects for cocoa tree plot yield estimation was the performance sensitivity to data from different networks. Two validation sets did not provide certainty, only trends. With a sensitivity of less than 0.5%, YOLOv8n took the lead in the top three.

With a dispersion of <0.007 s, the average CPU times per image between the different networks were considered to be similar: this slight fluctuation clearly had no impact on the cocoa pod detection workflow. With a dispersion of 0.07 s, the average GPU times per image reflected the impact of variations in the complexity of the architectures of the different networks studied. The GPU time was a complementary network classification indicator to choose the best trained network to implement in the cocoa pod counting workflow. With average GPU times per image 15–20% higher than those of the YOLOv8 and YOLOv11 networks, the RTDetr large and extra-large architectures were discarded even though their average cocoa pod detection capacity was just 2% lower than that of YOLOv8n. As the persistent and temporary data storage sizes were easy to evaluate according to the different neural architectures, the trends that emerged from our preliminary study should help establish guidelines for future studies to define a low-carbon footprint tool for early yield estimation in cocoa plantations.

## References

[1] Olofintuyi SS, Olajubu EA, Olanike D. An ensemble deep learning approach for predicting cocoa yield. Heliyon. 2023;9(4):e15245. doi: 10.1016/j.heliyon.2023.e15245 37089327 PMC10113837

[2] Kongor JE, Owusu M, Oduro-Yeboah C. Cocoa production in the 2020s: Challenges and solutions. CABI Agric Biosci. 2024. doi: 10.1186/s43170-024-00310-6

[3] Alain BK. Economic impact of cocoa culture in Ivory Coast and in Ghana from 1980 to 2015. J His Arch & Anthropol Sci. 2024;9(2):62–7.

[4] Gongal A, Amatya S, Karkee M, Zhang Q, Lewis K. Sensors and systems for fruit detection and localization: A review. Computers and electronics in agriculture. 2015;116:8–19.

[5] "Girshick R, Donahue J, Darrell T, Malik J. Rich feature hierarchies for accurate object detection and semantic segmentation. 2014 IEEE Conference on Computer Vision and Pattern Recognition, 2014. 580–7. 10.1109/cvpr.2014.81

[6] "Hou L, Wu Q, Sun Q, Yang H, Li P. Fruit recognition based on convolution neural network. 2016 12th International Conference on Natural Computation, Fuzzy Systems and Knowledge Discovery (ICNC-FSKD), 2016. 18–22. 10.1109/fskd.2016.7603144

[7] Rahnemoonfar M, Sheppard C. Deep count: Fruit counting based on deep simulated learning. Sensors (Basel). 2017;17(4):905. doi: 10.3390/s17040905 28425947 PMC5426829

[8] Xiao F, Wang H, Xu Y, Zhang R. Fruit detection and recognition based on deep learning for automatic harvesting: An overview and review. Agronomy. 2023;13(6):1625. doi: 10.3390/agronomy13061625

[9] Borianne P, Sarron J, Borne F, Faye E. Deep mango cultivars: Cultivar detection by classification method with maximum misidentification rate estimation. Precision Agric. 2023;24(4):1619–37. doi: 10.1007/s11119-023-10012-0

[10] Daviet B, Fournier C, Cabrera-Bosquet L, Simonneau T, Cafier M, Romieu C. Ripening dynamics revisited: An automated method to track the development of asynchronous berries on time-lapse images. Plant Methods. 2023;19(1):146. doi: 10.1186/s13007-023-01125-8 38098093 PMC10720176

[11] Sa I, Ge Z, Dayoub F, Upcroft B, Perez T, McCool C. DeepFruits: A fruit detection system using deep neural networks. Sensors (Basel). 2016;16(8):1222. doi: 10.3390/s16081222 27527168 PMC5017387

[12] Borianne P, Borne F, Sarron J, Faye É. Deep mangoes: from fruit detection to cultivar identification in colour images of mango trees. 2019. doi: 10.48550/arXiv.1909.10939

[13] Wan S, Goudos S. Faster R-CNN for multi-class fruit detection using a robotic vision system. Computer Networks. 2020;168:107036. doi: 10.1016/j.comnet.2019.107036

[14] Mirhaji H, Soleymani M, Asakereh A, Abdanan Mehdizadeh S. Fruit detection and load estimation of an orange orchard using the YOLO models through simple approaches in different imaging and illumination conditions. Computers and Electronics in Agriculture. 2021;191:106533. doi: 10.1016/j.compag.2021.106533

[15] Cheng R. A survey: Comparison between Convolutional Neural Network and YOLO in image identification. J Phys: Conf Ser. 2020;1453(1):012139. doi: 10.1088/1742-6596/1453/1/012139

[16] Sharma A, Kumar V, Longchamps L. Comparative performance of YOLOv8, YOLOv9, YOLOv10, YOLOv11 and Faster R-CNN models for detection of multiple weed species. Smart Agricultural Technology. 2024;9:100648. doi: 10.1016/j.atech.2024.100648

[17] Khanam R, Asghar T, Hussain M. Comparative performance evaluation of YOLOv5, YOLOv8, and YOLOv11 for solar panel defect detection. Solar. 2025;5(1):6. doi: 10.3390/solar5010006

[18] Raj R, Nagaraj SS, Ritesh S, Thushar TA, Aparanji VM. Fruit classification comparison based on CNN and YOLO. IOP Conf Ser: Mater Sci Eng. 2021;1187(1):012031. doi: 10.1088/1757-899x/1187/1/012031

[19] Jrondi Z, Moussaid A, Hadi MY. Exploring End-to-End object detection with transformers versus YOLOv8 for enhanced citrus fruit detection within trees. Systems and Soft Computing. 2024;6:200103. doi: 10.1016/j.sasc.2024.200103

[20] Safre ALS, Torres-Rua A, Black BL, Young S. Deep learning framework for fruit counting and yield mapping in tart cherry using YOLOv8 and YOLO11. Smart Agricultural Technology. 2025;11:100948. doi: 10.1016/j.atech.2025.100948

[21] Mamadou D, Ayikpa KJ, Ballo AB, Kouassi BM. Cocoa pods diseases detection by mobilenet confluence and classification algorithms. IJACSA. 2023;14(9). doi: 10.14569/ijacsa.2023.0140937

[22] Vera D, Oviedo B, Casanova WC, Zambrano-Vega C. Deep learning-based computational model for disease identification in cocoa pods (Theobroma cacao L.). 2024. doi: 10.48550/arXiv.2401.01247

[23] "Ayubi A, Faiz M, Situmorang GB, Ramadhani KN, Utama NP. A Cocoa Ripeness Detection and Classification Model Based on Improved YOLOv5s. 2023 10th International Conference on Advanced Informatics: Concept, Theory and Application (ICAICTA), 2023. 1–6. 10.1109/icaicta59291.2023.10390119

[24] Sykes JR, Denby KJ, Franks DW. Computer vision for plant pathology: A review with examples from cocoa agriculture. Appl Plant Sci. 2023;12(2):e11559. doi: 10.1002/aps3.11559 38638617 PMC11022223

[25] Ayikpa KJ, Mamadou D, Sodjinou SG, Ballo AB, Gouton P, Adou KJ. Detecting and extracting cocoa pods in the natural environment using deep learning methods. Lecture Notes in Networks and Systems. Springer Nature Switzerland. 2023. 164–74. doi: 10.1007/978-3-031-29857-8_17

[26] Lammoglia SKD, Borianne P, Théveny F, Cabrera-Bosquet L. Real-time image detection of cocoa pods in natural environment using deep learning algorithms. ICCO. 2023.

[27] Ren S, He K, Girshick R, Sun J. Faster r-cnn: Towards real-time object detection with region proposal networks. Advances in Neural Information Processing Systems. 2025;28:91–9.

[28] Liu Y, Lu B, Peng J, Zhang Z. Research on the use of YOLOv5 object detection algorithm in mask wearing recognition. World Sci Res J. 2020;6(11):276–84.

[29] Reis D, Kupec J, Hong J, Daoudi A. Real-time flying object detection with YOLOv8. 2023. doi: 10.48550/arXiv.2305.09972

[30] Khanam R, Hussain M. YOLOv11: An overview of the key architectural enhancements. 2024. doi: 10.2410.17725

[31] Carion N, Massa F, Synnaeve G, Usunier N, Kirillov A, Zagoruyko S. End-to-end object detection with transformers. Lecture Notes in Computer Science. Springer International Publishing. 2020. 213–29. doi: 10.1007/978-3-030-58452-8_13

[32] "Zhao Y, Lv W, Xu S, Wei J, Wang G, Dang Q, et al. DETRs Beat YOLOs on Real-time Object Detection. 2024 IEEE/CVF Conference on Computer Vision and Pattern Recognition (CVPR), 2024. 16965–74. 10.1109/cvpr52733.2024.01605

[33] Jegham N, Koh CY, Abdelatti M, Hendawi A. Evaluating the evolution of yolo (you only look once) models: A comprehensive benchmark study of yolo11 and its predecessors. 2024. doi: 10.48550/arXiv.2411.00201

[34] Arkin E, Yadikar N, Xu X, Aysa A, Ubul K. A survey: Object detection methods from CNN to transformer. Multimed Tools Appl. 2022;82(14):21353–83. doi: 10.1007/s11042-022-13801-3

[35] "He K, Zhang X, Ren S, Sun J. Deep Residual Learning for Image Recognition. 2016 IEEE Conference on Computer Vision and Pattern Recognition (CVPR), 2016. 770–8. 10.1109/cvpr.2016.90

[36] Oddoye EOK, Agyente-Badu CK, Gyedu-Akoto E. Cocoa and its by-products: Identification and utilization. Chocolate in Health and Nutrition. Humana Press. 2012. 23–37. doi: 10.1007/978-1-61779-803-0_3

[37] Yao Sadaiou Sabas B, Gislain Danmo K, Akoua Tamia Madeleine K, Jan B. Cocoa production and forest dynamics in ivory coast from 1985 to 2019. Land. 2020;9(12):524. doi: 10.3390/land9120524

[38] Dago MR, Zo‐Bi IC, Konan IK, Kouassi AK, Guei S, Jagoret P, et al. What motivates West African cocoa farmers to value trees? Taking the 4 W approach to the heart of the field. People and Nature. 2024;7(1):215–30. doi: 10.1002/pan3.10754

[39] Lammoglia SKD, Cabrera-Bosquet L, Rojas-Bustos JP. https://www.zooniverse.org/projects/phenoarch/cocoa-fruit-counting. 2022.

[40] "Simpson R, Page KR, De Roure D. Zooniverse: Observing the world’s largest citizen science platform. Proceedings of the 23rd international conference on world wide web, 2014. 1049–54.

[41] Abotsi KE, Arthaud G, Balembois E, Brou JM, Vaultier LC, Daubrey M, et al. Guide global pour la mise en œuvre d’une agroforesterie cacaoyère durable. Biodiversity and Conservation. 2022;15:4097–117.

[42] Sarron J. Estimation spatialisée des rendements d’une culture pérenne en Afrique de l’Ouest: le cas du manguier au Sénégal. Montpellier SupAgro. 2019.

[43] Kadhim ZS, Abdullah HS, Ghathwan KI. Artificial neural network hyperparameters optimization: A survey. Int J Online Biomed Eng. 2022;18(15):59–87.

[44] Sharma A, Kumar V, Longchamps L. Comparative performance of YOLOv8, YOLOv9, YOLOv10, YOLOv11 and Faster R-CNN models for detection of multiple weed species. Smart Agricultural Technology. 2024;9:100648. doi: 10.1016/j.atech.2024.100648

[45] "Rana PB, Thapa S. Comparative Study of Object Detection Models for Fresh and Rotten Apples and Tomatoes: Faster R-CNN, DETR, YOLOv8, and YOLOv12S. 2025 International Conference on Inventive Computation Technologies (ICICT), 2025. 660–7.

[46] Singh N, Tewari VK, Biswas PK. Vision transformers for cotton boll segmentation: Hyperparameters optimization and comparison with convolutional neural networks. Industrial Crops and Products. 2025;223:120241. doi: 10.1016/j.indcrop.2024.120241

[47] Rahman MA, Wang Y. Optimizing intersection-over-union in deep neural networks for image segmentation. Lecture Notes in Computer Science. Springer International Publishing. 2016. 234–44. doi: 10.1007/978-3-319-50835-1_22

[48] "Yacouby R, Axman D. Probabilistic Extension of Precision, Recall, and F1 Score for More Thorough Evaluation of Classification Models. Proceedings of the First Workshop on Evaluation and Comparison of NLP Systems, 2020. 79–91. 10.18653/v1/2020.eval4nlp-1.9

[49] Borianne P, Théveny F, Bertrand B, Villain L, Faye É, Sarron J, et al. L’IA au service de l’agriculture: au cœur de l’expérience Agro’Deep. 2021. https://hal.archives-ouvertes.fr/hal-05019239

[50] Kurtzer GM, Sochat V, Bauer MW. Singularity: Scientific containers for mobility of compute. PLoS One. 2017;12(5):e0177459. doi: 10.1371/journal.pone.0177459 28494014 PMC5426675

[51] Bailly A, Blanc C, Francis É, Guillotin T, Jamal F, Wakim B, et al. Effects of dataset size and interactions on the prediction performance of logistic regression and deep learning models. Comput Methods Programs Biomed. 2022;213:106504. doi: 10.1016/j.cmpb.2021.106504 34798408

[52] Dawson HL, Dubrule O, John CM. Impact of dataset size and convolutional neural network architecture on transfer learning for carbonate rock classification. Computers & Geosciences. 2023;171:105284. doi: 10.1016/j.cageo.2022.105284

[53] Borianne P. Agro’Deep project: the object annotation tools. 2026.

[54] "Trivedi J, Shah V, Tripathi P, Sasidhar K. Smartphones for computing: can they reduce the carbon footprint?. 2023 IEEE 11th Region 10 Humanitarian Technology Conference (R10-HTC). 2023. 196–200.

